# Medically Attended Injuries among Slovak Adolescents: Relationships with Socio-Economic Factors, Physical Fighting, and Physical Activity

**DOI:** 10.3390/ijerph17186721

**Published:** 2020-09-15

**Authors:** Peter Bakalár, Katarína Rosičová

**Affiliations:** 1 Department of Sports Educology and Humanistics, Faculty of Sports, University of Presov, 08001 Presov, Slovakia; 2 Department of Regional Development, Land-use Planning and Environment, Kosice Self-Governing Region, 04001 Kosice, Slovakia; katarina.rosicova@vucke.sk

**Keywords:** medically attended injuries, adolescents, family affluence, registered unemployment rate, average nominal monthly earning of employee, physical fighting, physical activity, Slovakia

## Abstract

There is a worrisome increase in the reporting of medically attended injuries in Slovak adolescents. The aim of this study is to examine the relationships between socio-economic factors, physical fighting, and physical activity with frequency of medically attended injuries among this population group. Data from 8902 adolescents participating in the Health Behavior in School-Aged Children study were used (mean age 13.37; 50.9% boys). The effects of family affluence, registered unemployment rate, average nominal monthly earnings of employees, physical fighting, and physical activity on frequency of medically attended injury were explored using linear regression analysis. Pearson’s correlation was used to describe the associations between all selected variables. The selected model of linear regression explained 15.8% of the variance in the frequency of medically attended injuries. All variables except the registered unemployment rate showed linear positive relationships with medically attended injuries. The correlation analysis confirmed linear positive associations between medically attended injuries and physical fighting, family affluence, physical activity, and average nominal monthly earnings of employees. Further research on these variables is needed in the Slovak context. This may include analyses of the nature of the relationships between socio-economic factors and medically attended injuries, as well as systematic evaluation of applied physical fighting and physical-activity-related injury interventions to support evidence-based policy making.

## 1. Introduction

Injury is a significant cause of death and morbidity among children from the age of one, which increases to become the leading cause of death among children aged 10 to 19 years [1]. In addition to death, tens of millions of children require hospital care for nonfatal injuries [2]. The etiology of adolescent injury is, therefore, a health research priority [3]. Various risk and protective factors of injuries were identified in school-aged children and adolescents at the individual and family levels, as well as for the level of the environment in which the child lives [4]. On the individual level, participation in physical activity is the main cause of unintentional adolescent injuries in many developed countries [5]. Even though deaths from participation in physical activity are uncommon, children and adolescents with nonfatal injuries may experience serious health consequences for the rest of their lives, especially those who injure their neck, spine, and joints (such as knees), or who suffer traumatic brain injuries [6]. In addition to the physical and financial costs, injured individuals may experience negative psychological consequences, including mood disturbances and lowered self-esteem, frustration, anger, daily living activity consequences (sleep disturbances, study issues), and feelings of loneliness, self-blame, or self-criticism [7,8].

Another factor that is consistently shown to cause injury on the individual level is physical fighting [9]. Physical fighting is a common manifestation of interpersonal violence observed in adolescent populations [10]. Because it is highly visible and often results in contact with health professionals, researchers have proposed fighting behavior as one of the earliest and most reliable markers of multiple-risk behavior syndrome, which can include substance use, truancy, and other problem behaviors during adolescence [11]. Children who fight report lower life satisfaction, poorer family and peer relationships, and worse perceptions of their school environments than do children not involved in fighting [12].

On the family level, the association between socio-economic status (SES) and increased risk of injury occurrence in school-aged children and adolescents is known from descriptive epidemiology, with lower SES associated with increased risk for hospitalization and fighting injury and higher SES associated with increased risks for physical activity injury [4,13]. Most recently, strong social inequalities were observed in adolescents’ injury prevalence, with adolescents from more affluent families more likely to receive medical treatment for injuries [14]. 

Based on data from the Health Behavior in School-Aged Children (HBSC) study, there is a worrisome rise in the reporting of medically attended injuries in Slovak adolescents between the years 2010 and 2018 [15], which creates an obvious need to focus on this problem in the Slovak context. Since the development and implementation of effective intervention programs requires a knowledge of the risk and protective factors for childhood injury [4], the aim of this study is to examine the relationships between socio-economic factors, physical fighting, and physical activity with frequency of medically attended injuries among this population group in Slovakia. Based on the literature review, we hypothesized positive relationships between physical fighting, physical activity, selected socio-economic variables (family affluence and average nominal monthly earnings of employees), and medically attended injuries. Subsequently, for the registered unemployment rate, we hypothesized a negative relationship with medically attended injuries.

## 2. Methods 

### 2.1. Sample and Procedure

Data were obtained from the World Health Organization collaborative HBSC study conducted in 2018 in Slovakia. The HBSC study used a two-step sampling procedure to obtain a representative sample. In the first step, 140 larger and smaller elementary schools located in rural and urban areas from all regions of Slovakia were asked to participate. These were randomly selected from a list of all eligible schools in Slovakia obtained from the Slovak Institute of Information and Prognosis for Education. The school response rate (RR) was 77.85%. In the second step, data from 8902 adolescents from the fifth to ninth grades of elementary schools in Slovakia were obtained (mean age 13.37; 50.9% boys). Since the adolescents did not answer all questions, the resulting samples of epidemiological data sets differ in number.

### 2.2. Ethical Statement

The study was approved by the Ethics Committee of the Medical Faculty at the Pavol Jozef Safarik University in Kosice (approval code: 16N/2017). Parents were informed about the study via school administration and could opt out if they disagreed with their child’s participation. Participation in the study was fully voluntary and anonymous, with no explicit incentives provided for participation.

### 2.3. Variables

The selected variables for analyses were chosen in light of observed social inequalities in adolescents’ injury prevalence and the consistent associations with physical fighting and physical activity. They consisted of HBSC questionnaire items (medically attended injuries, family affluence, physical fighting, and physical activity) and macroeconomic indicators (registered unemployment rate and average nominal monthly earnings of employees).

#### 2.3.1. Medically Attended Injuries

The HBSC item measuring the frequency of medically attended injury originates from the 1988 Child Health Supplement to the United States National Health Interview Survey, with the same item being regularly used in the Youth Risk Behavior Surveillance System (YRBS) [16]. It has been used in the HBSC survey since 1993/94 and is considered the standard item for studying injuries, having been substantially validated as part of the YRBS study and used in Canada [16,17]. The item and its response categories are as follows:

*During the past 12 months, how many times were you injured and had to be treated by a doctor or nurse?* (I was not injured in the past 12 months; 1 time; 2 times; 3 times; 4 times or more).

#### 2.3.2. Family Affluence

Family affluence was measured by the Family Affluence Scale (FAS version III) developed by the HBSC study as an alternative tool to parental occupational social class to increase the thoroughness and detail of research into social inequalities in health, and because many children, especially younger ones, have difficulty describing parental occupation. The validity of the FAS has been addressed by several studies [18]. The FAS III used in Slovakia consists of 6 items based on joint assessment and validation from the HBSC FAS development project [19]. The questions include new and refined items from previous FAS versions—bedrooms (FAS II), computers (FAS II), cars (FAS II), holidays abroad (refined), dishwasher (new), bathroom (new)—as defined by the HBSC 2013/2014 survey protocol [20]. The items and their response categories are as follows:

1. Does your family own a car or another motorized vehicle? (No = 0; Yes, one = 1; Yes, two = 2).
2. Do you have your own bedroom? (No = 0; Yes = 1).
3. How many computers (including laptops and tablets, not including game consoles and smartphones) does your family own? (None = 0, One = 1; Two = 2; More than two = 3).
4. How many bathrooms (room with a bath/shower or both) are there in your home? (None = 0; One = 1; Two = 2; More than two = 3).
5. Does your family have a dishwasher? (No = 0; Yes = 1).
6. How many times did you and your family travel out of the Slovakia for holiday/vacation last year? (Never = 0; Once = 1; Twice = 2; More than twice = 3).

The responses to the items were calculated as an aggregated FAS index ranging from 0 to 13. The index scores were then used to identify groups of young people in the lowest 20% (low affluence), middle 60% (medium affluence), and highest 20% (high affluence).

#### 2.3.3. Physical Fighting

The frequency of physical fighting is assessed in the HBSC study as a measure of aggression and violence and a component of multiple problem and risk behaviors. The frequency of fighting has been well validated and the reliability has been ascertained with extensive use in the United States YRBS [21]. The item and its response categories are as follows:

*During the past 12 months, how many times were you in a physical fight?* (I have not been in a physical fight in the past 12 months; 1 time; 2 times; 3 times; 4 times or more).

#### 2.3.4. Physical Activity

Physical activity was measured by an item adapted for use in the HBSC study from the item developed by Prochaska et al. [22] for the purposes of clinical practice with adolescents. The authors validated it against seven-day continuous measurement using an accelerometer (r = 0.40, *p* < 0.001) and observed its substantial test–retest stability (intraclass correlation coefficient (ICC) = 0.77). Similarly, the test–retest stability was found to be acceptable in the samples of Finnish (ICC = 0.6–0.8); Chinese (ICC = 0.82); and Czech, Slovak, and Polish (ICC = 0.6) 11–15-year-olds [23,24,25]. Moreover, the authors of an Australian study concluded that the self-reported moderate-to-vigorous physical activity (MVPA) index had an acceptable validity for measuring noncompliance with physical activity recommendations in 15–17-year-old adolescents. Comparing five days of valid accelerometer wearing time, the specificity for meeting current MVPA guidelines assessed by the MVPA index ranged from 60.8% (for boys) to 79.7% (girls) [26]. The item and its response categories are as follows:

*Over the past 7 days, on how many days were you physically active for a total of at least 60 min per day? Please add up all the time you spent in physical activity each day* (0 days; 1 day; 2 days; 3 days; 4 days; 5 days; 6 days; 7 days).

#### 2.3.5. Macroeconomic Indicators

The registered unemployment rate and average nominal monthly earnings of employees were used as macroeconomic indicators and were calculated for each district of Slovakia as an average for the period of 2016–2018. Subsequently, district averages were assigned to each adolescent based on the affiliation of their school to the corresponding district.

The registered unemployment rate was expressed as the proportion of the number of disposable job applicants to the total number of economically active individuals and was obtained from the tally performed by the Center of Labor, Social Affairs, and Family of the Slovak Republic. The mean values per district in the period of 2016–2018 were 7.2% (ranging from 2.4 to 19.7%).

The average nominal monthly earnings of employees was based on the data of the Statistical Office of the Slovak Republic, containing data on the wages for all companies and organizations in Slovakia, except the employees of self-employed persons. The mean monthly income in the districts of Slovakia was €976 in the period of 2016–2018 (ranging from €700 to €1535).

### 2.4. Statistical Analysis

The effects of family affluence, registered unemployment rate, average nominal monthly earnings of employees, physical fighting, and physical activity on the frequency of medically attended injuries were explored using linear regression analysis. The crude effect of all factors was included into the final model. Analyses were done for whole samples and separately for boys and girls. The regression models were checked for collinearity. Pearson’s correlation was used to describe the associations between all selected variables. Missing data were automatically excluded from the analyses.

Analyses were done using SPSS version 21.0 (IBM, New York, NY, USA).

## 3. Results

### 3.1. Epidemiological Data of the Sample

Medically attended injury was reported by 48.7% of Slovak adolescents, with higher prevalence in boys (51%) than girls (46.4%) (Table 1). A higher representation in the high family affluence category was identified in both boys and girls, compared to middle and low family affluence categories (Table 2).

Physical fighting was more prevalent in boys (42.6%) than in girls (19.8%). Overall, 31.2% of Slovak adolescents reported having been in physical fight 1 and more times during the last 12 months (Table 3). Boys also reported more frequently than girls to be physically active for 7 days, and in doing so meeting the physical activity recommendations for adolescents (Table 4).

### 3.2. Linear Regression Model

The selected model of linear regression consisted of socio-economic variables (family affluence, registered unemployment rate, and average nominal monthly earnings of employees), frequency of physical activity, and physical fighting explained 15.8% of the variance in the frequency of medically attended injuries. The strongest linear positive relationship was for frequency of physical fighting (*p* < 0.001), followed by family affluence (*p* < 0.001), frequency of physical activity (*p* < 0.001), and average nominal monthly earnings of employees (*p* < 0.05). The relationship between the registered unemployment rate and frequency of medically attended injuries was found to be nonsignificant (Table 5).

Among boys, the selected model explained 18% and among girls 13.6% of the variance in the frequency of medically attended injuries, while the relationships between the frequency of physical fighting, frequency of physical activity, family affluence, and frequency of medically attended injuries were stronger in boys than in girls. The relationships with the registered unemployment rate and average nominal monthly earnings of employees were nonsignificant both in boys and girls (Table 6).

### 3.3. Correlation Analysis

The correlation analysis confirmed the linear positive associations between the frequency of medically attended injuries and frequency of physical fighting (r = 0.328, *p* < 0.001), family affluence (r = 0.258, *p* < 0.001), frequency of physical activity (r = 0.155, *p* < 0.001), and average nominal monthly earnings of employees (r = 0.047, *p* < 0.001). The association with the registered unemployment rate was found to be negative but nonsignificant. The frequency of physical fighting was neither associated with the registered unemployment rate, nor the average nominal monthly earnings of employees. Only family affluence and physical activity were shown to be associated will all studied variables (Table 7).

## 4. Discussion

This study brings the evidence of the relationships between medically attended injuries in Slovak adolescents and family affluence, average nominal monthly earnings of employees, physical fighting, and physical activity. Therefore, we accept our hypotheses about the positive relationships between physical fighting, physical activity, selected socio-economic variables (family affluence and average nominal monthly earnings of employees), and medically attended injuries. On the other hand, the study does not support the relationship with registered unemployment rate, and therefore we reject this hypothesis.

The prevalence of medically attended injuries among Slovak adolescents was 48.7%, with higher prevalence in boys than girls. This prevalence was higher than the average prevalence in 45 countries and regions who participated in HBSC study in 2017/2018 and it was also higher compared to the results of the HBSC studies in Slovakia in 2013/2014 and 2009/2010 [15,27].

One of the explanatory factors of gender differences in medically attended injuries is societal gender inequality, which was recently positively associated with sex differences in adolescent injuries, physical fighting, and physical activity by de Looze et al. [28]. According to them, in all studied countries, boys reported more physical fighting, physical activity, and injuries than girls, but the magnitude of these sex differences varied greatly between countries. In more gender-unequal countries, boys reported higher levels of fighting and physical activity compared with boys in more gender equal countries. In girls, scores were consistently low for these outcomes; however, injury was more common in countries with less gender inequality. This could be an explanation for Slovakia as well. The gender inequality index (GII) declined between the years 2010, 2014, and 2018 (0.195, 0.186, and 0.190, respectively), with gender differences in medically attended injuries declining also (8.3%, 6.6%, and 4.6% respectively), however their prevalence in girls increased (26%, 30.7%, and 46.4% respectively) [15,29]. Since this study did not primarily aim to explain gender differences, future research should focus on this dynamic pattern and fill the knowledge gap regarding sex differences in adolescent health behaviors.

Among other variables, physical fighting seems to play a stronger role in the frequency of medically attended injuries in Slovak adolescents, with predictable gender differences being observed, since there were great differences in the frequencies of physical fighting between boys and girls. This finding is in line with previous research demonstrating that physical fighting along with substance use are the main predictors of injury in adolescents across many countries and in both genders [3,11,30,31]. The prevalence of physical fighting (31.2%), its association with medically attended injuries, and possible long-term consequences such as increased probability of violence in later life suggest that in Slovakia adolescent fights should not be undervalued and seen as a part of the growing process [32,33]. There can be various reasons why adolescents engage in fighting, such as self-defense, to gain or maintain respect, or anger, which can be addressed using various prevention strategies, including interventions that teach anger management and conflict resolution, that promote adolescent self-efficacy in using nonviolent strategies, and that address parental attitudes about fighting [34]. The effectiveness of such strategies and the search for other risk factors of this risk behavior should guide the future research in this largely neglected area of global health research [28,30,35,36].

Family affluence explained some of the variance of medically attended injuries and was associated with its frequency. The positive direction of the association indicates that the frequency of medically attended injuries increases with the higher family affluence category. As was concluded by Inchley et al. [14], this may reflect increased access to health services, as well as greater opportunities for physical activity in adolescents from higher affluence families. In this study, the frequency of physical activity was positively associated with family affluence and had some explanatory effect on the variance of medically attended injuries, with stronger explanatory effects in boys than in girls. At the same time, negative associations of medically attended injuries, family affluence, average monthly earnings of employees, and frequency of physical activity with registered unemployment rate, together with positive associations of average monthly earnings of employees, family affluence, and physical activity, may support the aforementioned conclusion.

Regarding the role of physical activity frequency in the frequency of medically attended injuries, the results are congruent with previous research findings relating the frequency of physical activity with a higher risk for physical-activity-related injuries in sports clubs and leisure time [5]. The literature also provides other sufficient examples of epidemiological studies that describe the incidence and burden of physical-activity-related injuries sustained during high-intensity sports, as well as during recommended types of lower intensity physical activity [37]. In a similar vein to the previously mentioned problem of physical fighting, the possible scale and long-term consequences of physical-activity-related injuries suggest that this topic should be subjected in Slovakia to more rigorous research in the future, especially in the context of the promotion of physical activity among children and adolescents as part of a healthy lifestyle [37].

The strength of this study is that data were collected from a large representative sample of Slovak adolescents using the rigorous Health Behavior in School-Aged Children study methodology. To the authors’ knowledge, this study was the first to examine the relationships between socio-economic factors, physical fighting, physical activity, and the frequency of medically attended injuries among a representative adolescent population in Slovakia. The cross-sectional nature of the study allowed the prevalence to be determined and for associations between selected variables to be identified. However, to draw causal relationships, cohort studies or randomized controlled studies are needed [38]. Additionally, the study did not include other risk or protective factors of medically attended injuries that could possibly affect the selected model.

## 5. Conclusions

The results of the study provide evidence of the relationships between medically attended injuries in Slovak adolescents and family affluence, average nominal monthly earnings of employees, physical fighting, and physical activity. The strongest relationship was found between medically attended injuries and physical fighting. Taking into account the high prevalence of physical fighting and its possible long-term consequences, it seems that more attention should be paid to this area of Slovak adolescents’ behavior. For instance, the focus should be on the systematic evaluation of applied interventions, in particular on their long-term impacts, the impacts on social factors, and cost-effectiveness [10]. Family affluence and average nominal monthly earnings of employees were both related to medically attended injuries. Given that the nature of these relationships is still unclear [9,14], further analyses are needed. On the other hand, the relationship of physical activity with medically attended injuries and the long-term consequences is clearer. Even though it is not realistic to expect the elimination of all physical-activity-related injuries, the majority of them are preventable [39]. Therefore, similar to physical fighting, the focus should be on the systematic evaluation of the applied interventions to support evidence-based policy making.

## Figures and Tables

**Table 1:** Frequency and prevalence of medically attended injuries among Slovak adolescents in the last 12 months.

	Not Injured in the Last 12 Months (%)	Injured 1 Time (%)	Injured 2 Times (%)	Injured 3 Times (%)	Injured 4 Times or More (%)
**Boys (*n* = 4016)**	1971 (49.0%)	1093 (27.2%)	493 (12.3%)	207 (5.2%)	252 (6.3%)
**Girls (*n* = 3937)**	2112 (53.6%)	985 (25.0%)	429 (10.9%)	180 (4.6%)	231 (5.9%)
**Total (*n* = 7953)**	4083 (51.3%)	2078 (26.1%)	922 (11.6%)	387 (4.9%)	483 (6.1%)

**Table 2:** Distribution of Slovak adolescents among family affluence categories.

	Low Family Affluence (%)	Middle Family Affluence (%)	High Family Affluence (%)
**Boys (*n* = 3251)**	966 (29.7%)	966 (29.7%)	1319 (40.6%)
**Girls (*n* = 3386)**	1072 (31.7%)	1015 (30.0%)	1299 (38.3%)
**Total (*n* = 6637)**	2038 (30.7%)	1981 (29.8%)	2618 (39.5%)

**Table 3:** Frequency and prevalence of physical fighting among Slovak adolescents in the last 12 months.

	Not Been in Physical Fight in the Last 12 Months (%)	Been in Physical Fight 1 Time (%)	Been in Physical Fight 2 Times (%)	Been in Physical Fight 3 Times (%)	Been in Physical Fight 4 Times or More (%)
Boys (*n* = 3996)	2295 (57.4%)	772 (19.4%)	389 (9.7%)	156 (3.9%)	384 (9.6%)
Girls (*n* = 3932)	3154 (80.2%)	381 (9.7%)	153 (3.9%)	85 (2.2%)	159 (4.0%)
Total (*n* = 792)	5449 (68.7%)	1153 (14.6%)	542 (6.8%)	241 (3.0%)	543 (6.9%)

**Table 4:** Frequency and prevalence of physical activity (60 min a day) among Slovak adolescents in the past 7 days.

	0 Days (%)	1 Day (%)	2 Days (%)	3 Days (%)	4 Days (%)	5 Days (%)	6 Days (%)	7 Days (%)
Boys (*n* = 4436)	179 (4.0%)	239 (5.4%)	381 (8.6%)	550 (12.4%)	625 (14.1%)	720 (16.2%)	506 (11.4%)	1236 (27.9%)
Girls (*n* = 4274)	178 (4.2%)	277 (6.5%)	490 (11.5%)	692 (16.2%)	750 (17.5%)	699 (16.4%)	385 (9.0%)	803 (18.8%)
Total (*n* = 8710)	357 (4.1%)	516 (5.9%)	871 (10.0%)	1242 (14.3%)	1375 (15.8%)	1419 (16.3%)	891 (10.2%)	2039 (23.4%)

**Table 5:** Linear regression between frequency of medically attended injuries and selected variables.

Variables	Medically Attended Injuries (Frequency)	
	Standardized Coefficients (Beta)	Sig.
Unemployment rate (%)	0.021	0.100
Monthly earning (EUR)	0.025	0.046 *
Physical activity (frequency)	0.097	0.000 ***
Physical fighting (frequency)	0.283	0.000 ***
Family affluence (FAS index)	0.193	0.000 ***
R^2^/adjusted R^2^	0.158/0.158	

* *p* < 0.05; *** *p* < 0.001; R^2^—explained variance.

**Table 6:** Linear regression between frequency of medically attended injuries and selected variables—boys and girls separately.

Variables	Medically Attended Injuries (Frequency)			
	Boys		Girls	
	Standardized Coefficients (Beta)	Sig.	Standardized Coefficients (Beta)	Sig.
Unemployment rate (%)	0.033	0.054	0.005	0.787
Monthly earning (EUR)	0.015	0.384	0.036	0.052
Physical activity (frequency)	0.109	0.000 ***	0.089	0.000 ***
Physical fighting (frequency)	0.301	0.000 ***	0.263	0.000 ***
Family affluence (FAS index)	0.202	0.000 ***	0.176	0.000 ***
R^2^/adjusted R^2^	0.181/0.180		0.137/0.136	

* *p* < 0.05; *** *p* < 0.001; R^2^—explained variance.

**Table 7:** Pearson correlation analysis between selected variables.

Variables	Unemployment Rate (%)	Monthly Earning (EUR)	Physical Activity (Frequency)	Physical Fighting (Frequency)	Family Affluence (FAS Index)
Medically attended injuries (frequency)	−0.015	0.047 ***	0.155 ***	0.328 ***	0.258 ***
Unemployment rate (%)		−0.633 ***	−0.031 **	0.008	−0.099 ***
Monthly earning (EUR)			0.028 **	0.017	0.142 ***
Physical activity (frequency)				0.112 ***	0.136 ***
Physical fighting (frequency)					0.176 ***

*** Correlation is significant at the 0.001 level; ** correlation is significant at the 0.01 level.
